# Cytokine concentration across the stratum corneum in atopic dermatitis and healthy controls

**DOI:** 10.1038/s41598-020-78943-6

**Published:** 2020-12-14

**Authors:** Maja-Lisa Clausen, S. Kezic, C. M. Olesen, T. Agner

**Affiliations:** 1 Department of Dermatology, Bispebjerg Hospital, University of Copenhagen, 2400 Copenhagen, NV Denmark; 2 Academic Medical Center, Amsterdam, The Netherlands; 3 Department of Dermatology and Venerology, Bispebjerg Hospital, Copenhagen University, Nielsine Nielsens vej opgang 9, 2. sal, 2400 Copenhagen, Denmark

**Keywords:** Prognostic markers, Skin diseases, Translational research

## Abstract

Tape stripping is a promising technique for assessment of epidermal biomarkers in inflammatory skin diseases. However, to facilitate its implementation in the clinical practice, a thorough validation regarding sampling strategy is needed. Knowledge of biomarkers variation in concentration across stratum corneum is scarce. Therefore, this study aimed to assess the variability of cytokines across stratum corneum using tape stripping technique by consecutive application of 21 adhesive tapes (D-squame) to lesional and non-lesional skin from 15 patients with atopic dermatitis (AD) and 16 healthy controls. Concentration of cytokines (IL-1α, IL-1b, IL-5, IL-18, IFN-γ, CCL17, CCL22, CCL27, CXCL8, CXCL10, TNF-α, TSLP, VEGFA) was determined in five different depths, using multiplex immunoassay. Comparing tape 4 with tape 21, no cytokine changed significantly in concentration in AD lesional skin. In AD non-lesional skin a small decrease was found for CCL17, CXCL8 and CXCL10. For healthy controls, a decrease was found for IL-1a, IL-1b, VEGFA and an increase for IL-18. Differences were found between AD skin and healthy control skin. Concentration of cytokines was stable across stratum corneum, indicating that sampling of only one tape from the stratum corneum is reliable in reflecting the overall cytokine milieu. Differences between AD and healthy skin confirm robustness of tape stripping for measuring cytokine levels.

## Introduction

In recent years, tape stripping technique for collection of epidermal material for assessment of biomarkers has been introduced in research of inflammatory skin diseases, including atopic dermatitis (AD)^1–6^. Skin biopsies are often used for assessment of cutaneous biomarker expression^7–10^, however their invasive nature limits their use in children and infants, and furthermore hinder follow-up of previously sampled areas. Biopsies may induce pain, leave scars and pose a risk of infection, therefore there has been an immense focus on developing a minimally invasive sampling technique to allow for epidermal molecular profiling without the side effects of skin biopsies. The use of tape stripping for collecting stratum corneum material has thus been investigated and validated for evaluation of epidermal immune and barrier markers in both children and adults with AD^1–4,6,11^. A consistent method has been developed, using a specialized tape (D-squame tape), and standardized pressure^12,13^. Usually, between 20 and 35 tapes are applied consecutively to the skin, and stripped off gently. However, despite the effort to standardize this method, number of tape strips collected, and the specific tape number used for subsequent biomarker profiling, vary between studies. The use of tape stripping to collect stratum corneum has introduced the possibility of dividing stratum corneum in different layers, i.e. upper stratum corneum in the first 5–10 tapes, intermediate stratum corneum in tape 10–20, and lower stratum corneum in tape 20–35^14^. However, skin furrows create variations in the skin surface, and it has been discussed that one tape therefore might not reflect a single layer in stratum corneum^15^. Depth dependent concentration of epidermal biomarkers is sparsely investigated; however, we know the amount of total protein decreases with increasing depth in stratum corneum, yet the amount of soluble protein, including antimicrobial peptides and IL-1α, do not change across stratum corneum^12,14^.

AD is a common multifactorial disease, affecting up to 20% of children and 5% of adults^16^. A complex interplay between activation of a TH2/Th22-polarized immune response and skin barrier dysfunction plays a key role in the pathogenesis of AD^17^. In general, AD exhibits great heterogenicity in its molecular phenotype and is believed to comprise many subtypes (i.e., intrinsic/extrinsic, pediatric/adult, etc.)^18^. A need for understanding the molecular fingerprint of each subtype therefore is one of the necessary steps towards establishing a personalized medicine approach in the management of AD. Prior studies have shown that tape-strips capture the molecular phenotype of AD and other inflammatory skin diseases, and it is believed that molecular profiling of material from tape strips may be useful in the clinical setting for objectively tracking disease activity, and predicting disease course and treatment response. In order to establish the tape stripping method as an easy, reliable and useful alternative for skin biopsies, it is thus important to clarify whether the number of tapes and the depth reached influence expression of biomarkers. Therefore, the primary aim of the present study was to investigate changes in cytokine concentration across the stratum corneum in AD skin and healthy control skin. A secondary aim was to compare overall differences in cytokine concentration between skin of adult AD patients and adult healthy controls.

## Results

### Population and samples

Fifteen adult AD patients, all European Caucasian, were included in the study, nine women and six men. Mean age 36 (19–59) and mean O-SCORAD 34.7 (14.8–57.5). All patients used topical corticosteroids (TCS), though had not used treatment within 1-week before sampling. Sixteen adult healthy controls, all European Caucasian, were included in the study, 4 women and 12 men, with mean age 29 (24–59). Mean TEWL was significantly higher in AD patients (TEWL 13.5) compared to healthy controls (TEWL 9.4) (p-value 0.01), no marked difference in skin pH was found. Demographic and clinical data are given in Table 1.

**Table 1:** Demographic data.

	AD (n = 15)	HC (n = 16)
Female:male	9:6	4:12
Age; average (range)	36 (19–59)	29 (24–59)
Systemic treatment	8	0
**Topical treatment**		
Protopic	7	0
Corticosteroid	15	0
**Atopic disposition**	8	4
Asthma	8	0
Allergy	11	0
Hayfever	10	1
FLG mutation	4	0
**Skin barrier**		
TEWL; average (range)	13.4 (4–33.2)	9.5 (2.7–18.2)
Ph; average (range)	6.19 (4.9–7.08)	5.85 (4.97–6.89)
**Objective-SCORAD; average (range)**	34.7 (14.8–57.5)	NA
Mild	1	
Moderate	9	
Severe	5	

A total of 21 tapes were consecutively applied and stripped from the skin, and tape number 4, 6, 11, 16 and 21 (T4, T6, T11, T16, T21) were used for cytokine analysis. Hence five lesional skin samples (extremities and back) and five non-lesional skin samples (volar forearm) were included for each AD patient, and five samples from each healthy control (volar forearm), with a total of 160 samples from AD patients, and 80 samples from healthy controls.

**Figure 1:**
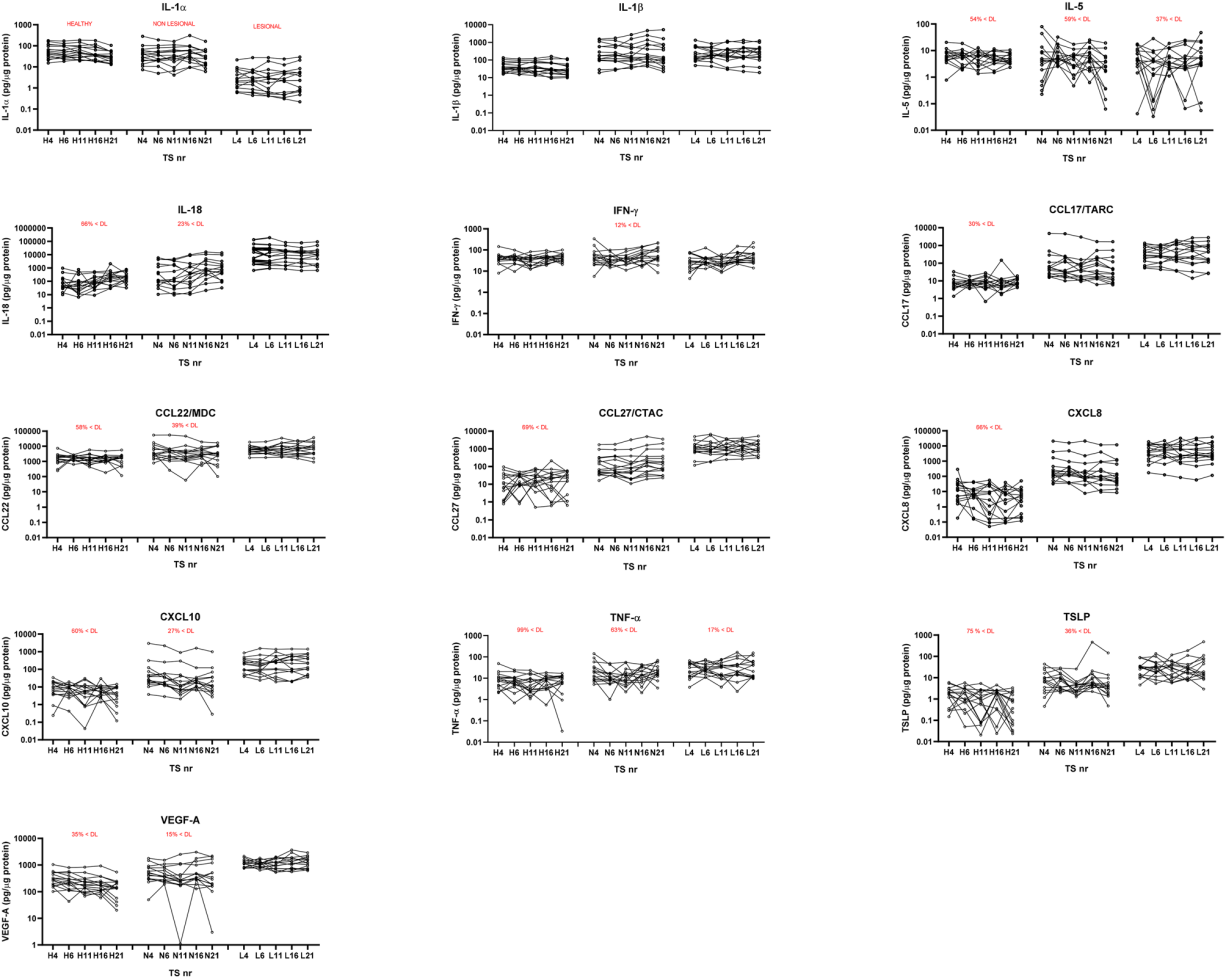
Concentration of cytokines across the stratum corneum in healthy controls (H), non-lesional skin of atopic dermatitis (N) and lesional skin of atopic dermatitis (L). *TS* tape strip number. A total of 13 different cytokines are determined on tape no 4, tape 6, tape 11, tape 16 and tape 21. The percentage of samples that were below detection limit is written in red: x < DL. Y-axis is a logarithmic scale. Concentrations of cytokines are un-adjusted and normalized to protein levels (pg/μg protein).

In AD patients, 9% of samples (10 LS samples and 4 NLS samples) were below the curve fit range for IL-5. In Healthy controls, 18% of samples for TSLP, 16% of samples for CCL27 and 20% of samples for IL-8 were below curve fit range. Since this was less than the 30% limit, all samples are included in the analyses (AD and healthy controls). The percentage of samples below detection range but within fit curve range is shown in Fig. 1 when more than 10% of samples were below detection range. Healthy control skin and AD non-lesional skin were more often in the low range compared to AD lesional skin.

### Changes in concentration through depth of stratum corneum

Comparing the concentration of the individual cytokines from the outer stratum corneum (T4) to the deeper stratum corneum (T21), no significant changes were found for AD LS. For AD NLS, a small but significant decrease with increasing depth was found for CCL17, CXCL8 and CXCL10 (corrected p-value < 0.05) (Table 2, Fig. 1). For healthy control skin, a significant decrease in concentration from T4 to T21 was found for IL-1α, IL-1β and VEGFA, and an increase in concentration was found for IL-18 (corrected p-value < 0.05) (Table 2). Cytokines characterised by significant changes in concentration, had a decrease in concentration at the deeper stratum corneum layers, except IL-18, which had an increase in the deeper layers (Fig. 1).

**Table 2:** Change in cytokine concentration trough depths of stratum corneum.

Cytokines	HC	AD NLS	AD LS
IL-1α	Yes T4,6,11, 16 vs T21	No	No
IL-1β	Yes T4, 6, 11 vs T21	No	No
IL-5	No	Yes T6 vs T21	No
IL-18	Yes T4, 6, 11vs T16,21	Yes T4, T6, T11 vs T16	Yes T4 vs T16
IFN-γ	No	Yes T6, 11 vs T21	Yes T11 vs T16,21
CCL17/TARC	No	Yes T4 vs T11,21	No
CCL22/MDC	No	No	No
CCL27/CTACK	No	Yes T11 vs T16, T21	No
CXCL8/IL-8	No	Yes T4, T6 vs T21	No
CXCL10/P-10	No	Yes T4,6 vs T11,21	No
TNF-α	No	No	No
TSLP	No	Yes T4 vs T11; T11 vs T16	No
VEGFA	Yes T4 vs T11,16,21; T6 vs T21	No	No

For each column (HC, AD NLS, AD LS) a comparison has been made for each depth towards every other depth, e.g.: T4 vs T4, T4 vs T6, T4 vs T11… T6 vs T11 and so forth. The comparisons with significant findings are written in the table e.g. T4 vs T21.

ANOVA multiple comparison for paired data, non-parametric analysis (Friedman’s test) has been performed, with correction for multiple testing controlling false discovery rate (FDR) using original FDR method by Benjamini and Hochberg. Significant findings with corrected p-value < 0.05 are marked as “Yes”.

*HC* healthy controls, *AD* atopic dermatitis, *NLS* non-lesional skin, *LS* lesional skin, *T4* Tape 4, *T6* Tape 6, *T11* Tape 11, *T16* Tape 16, *T21* Tape 21.

**Figure 2:**
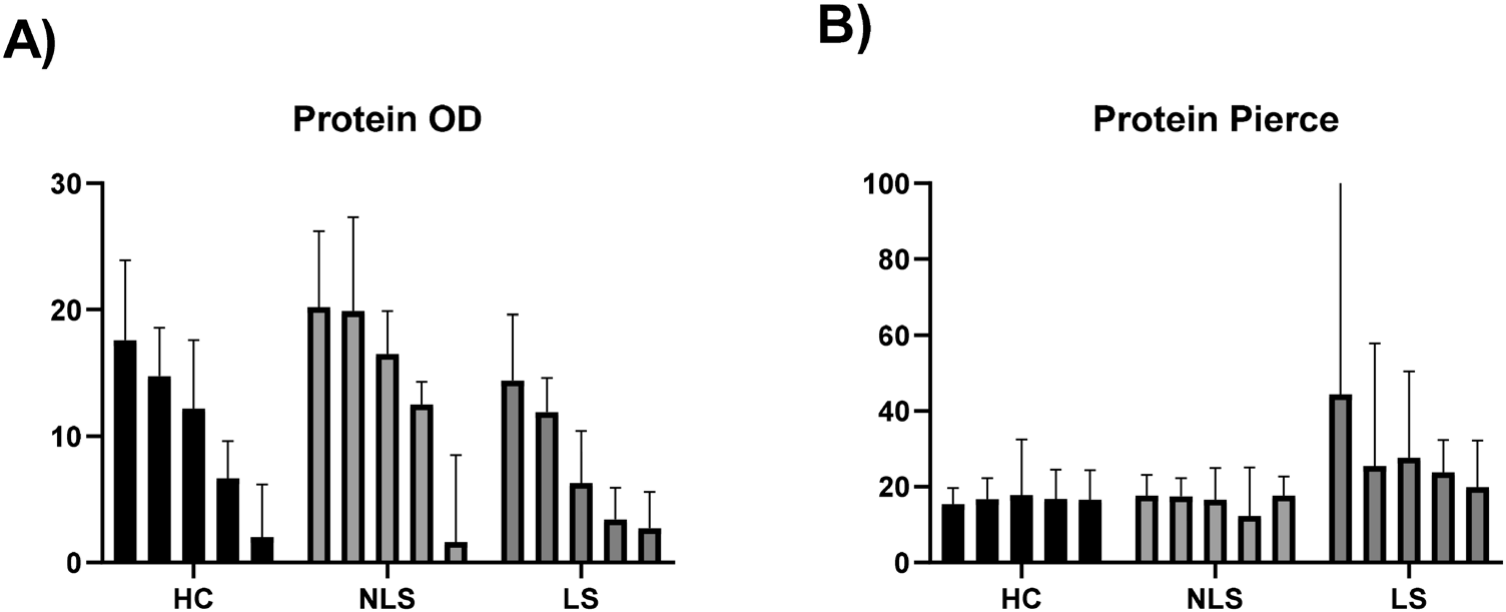
Protein content in tape strip samples of stratum corneum. (**A**) OD values measured by squame scan, showing decreasing amount of total protein through the depths of stratum corneum. (**B**) Soluble protein measured by Pierce BCA protein KIT, showing stable levels of protein across the depth of stratum corneum. Bars represent median with whiskers showing inter quartile range (IQR). The five columns represent tape no 4, tape no 6, tape no 11, tape no 16, tape no 21. *HC* healthy controls, *NLS* non lesional skin of atopic dermatitis patients, *LS* lesional skin of atopic dermatitis patients.

The amount of total protein, as evaluated by squame scan OD shows decreasing levels of total protein with increasing depth in stratum corneum. The amount of soluble protein, as measured by Pierce BCA protein kit, shows stable levels of protein through the depths of stratum corneum (Fig. 2).

### Interpersonal variation

Large variation was seen in cytokine concentration between individuals (Table S1). Inter-personal variation showed the lowest variation in healthy controls with a coefficient of variation (CV%) between 41 and 98%, and AD LS with a CV% between 35 and 142%, and the highest variation was found in AD NLS with a CV% between 57 and 295%.

### Differences between skin categories

Significant differences in cytokine concentration were found between AD skin and healthy control skin, and between AD NLS and AD LS. The only two cytokines that showed no difference between patients and controls were IL-5 and IFN-γ. Comparing mean concentrations, significant differences were found between AD LS and healthy control skin for IL-1α, IL-1β, IL-18, CCL17, CCL22, CCL27, CXCL8, CXCL10, TNF-α, TSLP, VEGFA and (corrected p-value < 0.001) (Table 3). Comparing AD NLS and healthy control skin, significant differences were found for IL-1β, CCL17, CCL22, CCL27, CXCL8, CXCL10, TNF-α, TSLP, VEGFA (corrected p-value < 0.05). Comparing AD LS with AD NLS, significant differences were found for IL-1α, IL-18 CCL17, CCL27, CXCL8, CXCL10, TNF-α, TSLP, VEGFA (corrected p-value < 0.05) (Table 3).

**Table 3:** Mean cytokine concentration in healthy controls and atopic dermatitis.

Cytokine	Concentration pg/μg protein (mean ± SD)			P-value
	HC	AD NLS	AD LS	
IL-1α	57.2 ± 43.3	49.7 ± 54.9	4.9 ± 6.8	HC-NLS: 0.43 **HC-LS: < 0.0001** **NLS-LS: < 0.0001**
IL-1β	43.9 ± 33.8	547 ± 926.4	331.7 ± 315.2	**HC-NLS: < 0.0001** **HC-LS: 0.0003** NLS-LS: 0.09
IL-5	6.1 ± 4.5	7.6 ± 11.3	6.9 ± 8.0	HC-NLS 1.0 HC-LS 0.5 NLS-LS 0.68
IL-18	210.3 ± 294.8	1814 ± 3226	20,383 ± 30,681	**HC-NLS: 0.045** **HC-LS: < 0.0001** **NLS-LS: < 0.0001**
IFN-γ	42.1 ± 21.9	57.2 ± 53.7	41.1 ± 34.7	HC-NLS 0.73 HC-LS 0.69 NLS-LS 0.17
CCL17/TARC	10.2 ± 16.5	283 ± 847.6	569.7 ± 603.8	**HC-NLS: 0.0005** **HC-LS: < 0.0001** **NLS-LS: 0.008**
CCL22/MDC	1756 ± 1212	6233 ± 10,298	8010 ± 6993	**HC-NLS: 0.009** **HC-LS: < 0.0001** NLS-LS: 0.15
CCL27/CTACK	25.6 ± 29.6	412.6 ± 850.4	1526 ± 1369	**HC-NLS: 0.002** **HC-LS: < 0.0001** **NLS-LS: 0.0009**
CXCL8/IL-8	14.7 ± 34.0	1481 ± 4201	6984 ± 8099	**HC-NLS: 0.0007** **HC-LS: < 0.0001** **NLS-LS: 0.002**
CXCL10/P-10	8.1 ± 7.0	148.2 ± 469.7	307.5 ± 350.3	**HC-NLS: 0.006** **HC-LS: < 0.0001** **NLS-LS: 0.015**
TNF-α	8.4 ± 7.0	21.6 ± 22.7	25.5 ± 31.4	**HC-NLS: 0.0007** **HC-LS: < 0.0001** **NLS-LS: 0.021**
TSLP	1.59 ± 1.4	15.5 ± 55.9	41.5 ± 62.6	**HC-NLS: 0.0009** **HC-LS: < 0.0001** **NLS-LS: 0.0067**
VEGFA	251.7 ± 201.2	609 ± 615.4	1265 ± 597.2	**HC-NLS: 0.016** **HC-LS: < 0.0001** **NLS-LS: 0.0002**

Bold letters indicate statistical significant p-values.

Mean concentration of each cytokine ± standard deviation (SD) within healthy control (HC) skin, atopic dermatitis (AD) non-lesional skin (NLS) and AD lesional skin (LS). Comparison between LS and NLS are made with Wilcoxon test. Comparison between HC and LS, and HC and NLS are made with Kruskal–Wallis and FDR Benjamini–Hochberg correction.

Comparing cytokine concentration at each depth (T4-T21), significant differences were also found between AD skin and healthy control skin, as well as AD LS and AD NLS (Table S2).

The overall tendency was that cytokine concentration was higher in AD LS compared to AD NLS and healthy control skin, however, for IL-1α, the opposite was seen, with the lowest concentration in AD LS (Fig. 1, Table 3). For soluble protein, a significant difference for mean protein content was found between AD LS and AD NLS (corrected p-value < 0.02) and between AD LS and HC (corrected p-value 0.03) (Fig. S3.)

## Discussion

This is the first study aimed to investigate whether selected protein biomarkers are differentially expressed across the stratum corneum. We evaluated the concentration of 13 selected cytokines (pg/μg total soluble protein) across the stratum corneum and found that the concentration is largely stable with no significant depth dependent gradient for the majority of investigated cytokines. This is in line with previous studies investigating antimicrobial peptides and IL-1α, showing no differential expression across the stratum corneum^14,19^.

Tape stripping is a promising skin sampling technique for use in clinical research as well as clinical trials to evaluate treatment efficacy^20,21^. It has the advantage of being non-invasive allowing for serial sampling in longitudinal studies and sampling in children. While skin biopsies remain superior for evaluating cutaneous histology and morphology, tape stripping is useful for a non-invasive assessment of the epidermal molecular phenotype. Thus, tape stripping has the potential to become a helpful tool in the clinic for analysis to differentiate between molecular subtypes of AD as part of a targeted and personalized treatment program. Other molecular components, including filaggrin, lipids and terminal differentiation products are all known to play an important role in AD and part of the molecular subtype, and have also been evaluated in tape strips^1,3,6,22^. Despite the increasing use of tape stripping, a standardized method for how many tape strips to sample and which tape number to use for subsequent analyses have not been evaluated adequately.

Up to 35 tapes are needed to remove the stratum corneum^23^, which causes a red, dry, slightly irritated patch, that may induce discomfort in some patients. Collecting 35 tapes is time consuming, but also labor intensive in the lab and expensive to process, thereby limiting the use in large-scale studies and in the clinic. The stable cytokine environment across the stratum corneum, demonstrated in the present study, allows for fewer tapes to be collected and enables comparison between studies in which different tape numbers are analyzed. Total amount of protein has previously shown to decrease with increasing depth in stratum corneum, whereas the amount of soluble protein is stable^13^ across the stratum corneum. We confirmed these data in the present study. Therefore, it is important to normalize the level of cytokines to the amount of soluble protein, as the measured cytokines are believed to originate from the extracellular space as also the soluble proteins do. Cytokines might be transferred to the extracellular space with the lipids which are secreted from the lamellar bodies on the boundary of the stratum granulosum and stratum corneum. This might also explain why there is no concentration gradient in cytokine expression across the stratum corneum. Previously, discussion on layers within the epidermis suggested that one tape does not reflect a single layer within stratum corneum due to skin furrows^15^. However, in light of the presented data in this study, and previous data on antimicrobial peptide expression^19^, it seems that stratum corneum might be viewed as one compartment in relation to the molecular milieu, thereby making skin barrier research by tape stripping easier to apply, since depth may not introduce any bias regarding the concentration of inflammatory markers.

Cytokines and chemokines are crucial signaling peptides secreted by resident skin cells (keratinocytes, dendritic cells, melanocytes and mast cells) as well as recruited immune cells (neutrophils, lymphocytes, eosinophils) forming the inflammatory infiltrates. Since the stratum corneum consists of dead and cornified cells (corneocytes), cytokines are expressed by living cells in the deeper epidermal and dermal layers. Accordingly, a higher concentration in the deeper stratum corneum might have been hypothesized, due to increased degradation of the cytokines or a diffusion gradient. However, we did not find this trend in this study. A likely explanation would be that the cytokines are transported upwards with the lipids within the stratum corneum, so a concentration gradient does not occur.

Overall and across the stratum corneum, AD skin as compared to control skin displayed increased expression of cytokines involved in innate immunity (IL-18, CXCL8, IL-1β, TNF-α), Th1- (CXCL10), Th2-signaling (CCL17, CCL22, CCL27, TSLP) and angiogenesis (VEGFA) paralleled by a decreased expression of IL-1α. This is in line with findings of previous tape studies in both children and adults with AD^2,4–6^. Additional, the majority of cytokines were increased in AD lesional skin compared to AD non-lesional skin, and further increased in AD non-lesional skin compared to healthy control skin. Thus, our data confirm the Th2-polarized immune activation in both lesional and non-lesional AD skin demonstrated in previous skin biopsy and tape strip studies^3,7,8,24–26^. Potential leakage of serum and hence serum proteins into the epidermal compartment in AD lesional skin due to a broken barrier cannot be ruled out, however this is a challenge not only in our study, but a general concern when investigating AD lesional skin. Nevertheless, previous studies have shown a better correlation between lesional and non-lesional compared to serum skin as well as greater immune dysregulation in the skin of AD patients, rather than serum^5,27^.

Concentration of IL-5 and IFN-γ did not differ between AD and healthy control skin, in contrast to previous data from skin biopsies^8,24^. Since tape strips collect only the superficial epidermis (mainly stratum corneum), these inconsistencies might be explained by differences in sampling techniques, or discrepancies between expression of mRNA (assessed in biopsy studies) and mature protein (assessed in tape strips). The lack of differential expression of IL-5, is in accordance with a recent tape strip study in infants with AD^5^, however, in contrast to a study by Koppes et al. that demonstrated increased expression of IL-5 and IFN-γ in tape strips obtained from patients with AD compared to healthy controls. Also in interstitial fluid from AD lesional skin^28^, elevated IL-5 concentration was found compared to healthy controls. These discrepancies are interesting and might be explained by the degree of severity at the local lesion or use of topical treatment.

We found a larger interpersonal variation in cytokine concentration between AD patients, larger than what was demonstrated for healthy controls, which is to be expected as the patients differed in disease severity and the samples were collected from different anatomical locations. The largest variation was seen within non-lesional skin of AD, which might reflect the subtle inflammatory changes reported to be found in non-lesional skin, despite no visual eczema, but also distance from the lesion plays an important role^29^. The finding that interpersonal variation in cytokine concentration was of much greater magnitude than the slight depth dependent variation revealed for the assessed cytokines, supports the heterogenic immunological phenotype of AD and emphasises the utility of tape strips profiling for molecular stratification of patients into endotypes.

A strength of the present study is the standardized method and collection of multiple tape strips from each participant. The diagnosis of AD was determined by a specialized dermatologist, and the majority of patients presented with moderate-severe AD. A limitation of the study is that only 13 protein biomarkers were assessed. Although the concentration of the evaluated markers did not change significantly, it cannot be ruled out that other biomarkers vary in stratum corneum expression pattern, and also a different pattern may be present when assessing mRNA.

In conclusion, tape strips have shown to be a valuable method for profiling different epidermal biomarkers and show great potential as a helpful tool for stratification of patients to different endotypes. Herein we provide valuable data for future studies using tape stripping for epidermal biomarker profiling, showing that the concentration of a panel of protein biomarkers are stable across the stratum corneum. Hence, our data suggest that only few tapes are needed for cytokine profiling and enables comparison between studies as stratum corneum depth does not seem to cause bias.

## Methods and materials

### Population

Patients were recruited from the outpatient clinic of the Department of Dermatology, Bispebjerg Hospital, from March-November 2017. Inclusion criteria were: AD according to UK-criteria, age ≥ 18 years, no treatment with phototherapy or systemic immunosuppressive drugs 4 months prior to sampling, and no use of topical anti-inflammatory treatment on sample location 7 days prior to sampling. Healthy controls were included. Inclusion criteria were age ≥ 18 years, and no history of AD or other inflammatory skin disease. The severity of AD was assessed using the Objective Scoring Atopic Dermatitis (O-SCORAD)^30^. All samples from healthy controls and non-lesional skin (NLS) samples from patients were taken from the volar forearm at least 7 cm from a lesion. Samples from lesional skin (LS) from patients were taken depending on location of the lesion (arm = 10, back = 3, foot = 2).

### Sampling of stratum corneum

The method was previously described^13^. In brief, stratum corneum was sampled by consecutive application of twenty-one adhesive D-Squame tapes (22 mm diameter, 3.8 cm^2^) (D-squame, CuDerm, Dallas TX, USA) onto the skin and pressed for 10 s with standardized pressure (225 g/cm^2^, D-Squame Pressure Instrument D500)^19^. Tape strips were placed individually in cryo-vials and immediately stored at − 80 °C. Measurements of transepidermal water loss (TEWL) and skin pH were measured on non-lesional skin, on the volar side of the distal forearm using the DermaLab open chamber Evaporimeter (Cortex Technology Hadsund, Denmark) and a skin-pH-meter (METTLER TOLEDO, Greisensee, Switzerland), respectively^19^.

### Cytokine analysis in tape strips

The 4th, 6th, 11th, 16th and 21st consecutive tape strip from each skin area were used to measure cytokine levels, as previously described^29^. To each vial 1.2 mL phosphate-buffered saline (Merck, Darmstadt, Germany) containing 0.005% Tween 20 (Sigma-Aldrich, Zwijndrecht, the Netherlands). Extraction of cytokines and soluble proteins was performed with an ultrasound sonication bath (Branson 5800, Branson Ultrasonics BV, The Netherlands) for 15 min in ice water. Extract aliquots of 200 μL were distributed in vials and stored at − 80 °C until further analysis. Concentrations of cytokines were determined using MESO QuickPlex SQ 120 (MSD, Rockville, MA, USA) according to the manufacturer’s instructions^29^.

The assessed cytokines were: IL-1α, IL-1β, IL-18, IL-5, IFN-γ, CCL17, CCL22, CCL27, CXCL8, CXCL10, TNF-α, TSLP, VEGFA (U-plex assays, MSD, Rockville, MA, USA).

Protein content on each tape was determined using squame scan (CuDerm, Dallas TX, USA) OD measures for total protein, as well as Pierce Micro BCA Protein Assay Kit (Thermo Fischer Scientific, Rockford, IL, USA) for soluble protein. Protein determination was carried out according to protocol given by the manufacturer. The amount of cytokine was standardized to the amount of soluble protein (BCA) on each tape. For statistical analysis, cytokine concentrations below the detection limit, but within fit curve range, were taken unchanged, and cytokine concentrations below the fit curve range were assigned half the value of the lowest sample concentration below the detection limit to maintain the ranking order. If more than 30% of the samples were below the curve fit range, the data for that cytokine will not be used (https://doi.org/10.1021/es071301c)^29^.

### Statistical analysis

Non-parametric One-Way ANOVA for multiple comparison were used. For comparison between skin categories AD vs HC, unpaired Kruskal–Wallis test was used, and for AD LS vs AD NLS, paired Friedman’s test was used. For comparing different depths within each skin category, paired Friedman’s test was used. Adjustment for multiple testing was performed using the original false discovery rate by Benjamini-Hochberg (α = 0.05). All comparisons are performed on raw cytokine concentration with no adjustments. Adjusted P-value < 0.05 was considered significant. Graph Pad Prism 8 was used for statistical calculations.

### Ethical approvals

The study was approved by the local science ethics committee ‘De Videnskabsetiske Komiteer for Region Hovedstaden’ (Protocol nr.: H-16047983) and the Danish Data Protection Agency (BFH-2017-042, I-Suite: 05449). Participants provided signed informed consent. All methods were carried out in accordance with relevant guidelines and regulations.

## Supplementary Information

Supplementary Information.
