# Supplementary material for: Cytokine concentration across the stratum corneum in atopic dermatitis and healthy controls
Source: Sci Rep. 2020 Dec 14;10:21895. doi: 10.1038/s41598-020-78943-6 (PMC7736354; doi:10.1038/s41598-020-78943-6)
Supplement: Supplementary file 1 — Supplementary Information. [file 41598_2020_78943_MOESM1_ESM.docx]

Cytokine concentration across the stratum corneum in atopic dermatitis and healthy controls - Supplementary files

M-L Clausen^1^, S Kezic^2^, C M Olesen^1^, T Agner^1^

^1^Department of Dermatology, Bispebjerg Hospital, University of Copenhagen, 2400 Copenhagen NV, Denmark

^2^Coronel Institute of Occupational Health, Amsterdam UMC, Amsterdam, the

Netherlands

Table S1: Inter-personal variation in cytokine concentration

| Cytokine | Interpersonal variation  CV% | | |
| --- | --- | --- | --- |
|  | HC | AD NLS | AD LS |
| IL-1α | 71 | 107 | 137 |
| IL-1β | 76 | 160 | 90 |
| IL-5 | 42 | 82 | 91 |
| IL-18 | 92 | 160 | 142 |
| IFN-γ | 41 | 57 | 67 |
| CCL17/TARC | 75 | 279 | 94 |
| CCL22/MDC | 60 | 150 | 76 |
| CCL27/CTACK | 81 | 193 | 76 |
| CXCL8/IL-8 | 98 | 281 | 94 |
| CXCL10/P-10 | 51 | 295 | 109 |
| TNF-α | 66 | 64 | 69 |
| TSLP | 61 | 222 | 96 |
| VEGFA | 73 | 94 | 35 |

Table S2: Significant differences between skin categories, at different depths

| Cytokines | HC vs AD NLS | HC vs AD LS | AD NLS vs AD LS |
| --- | --- | --- | --- |
| IL-1α | No | Yes***  All layers | Yes***  All layers |
| IL-1β | Yes**  All layers | Yes***  All layers | No |
| IL-5 | No | No | No |
| IL-18 | No | Yes***  All layers | Yes***  All layers |
| IFN-γ | No | No | No |
| CCL17/TARC | Yes*  All layers | Yes***  All layers | Yes*  All layers |
| CCL22/MDC | No | Yes***  All layers | No |
| CCL27/CTACK | Yes*  All layers | Yes***  All layers | Yes*  All layers |
| CXCL8/IL-8 | Yes*  All layers | Yes***  All layers | Yes **  All layers |
| CXCL10/P-10 | No | Yes***  All layers | Yes**  T11, T16, T21 |
| TNF-α | No | Yes**  All layers | Yes*  T6, T11 |
| TSLP | Yes*  T6, T16 | Yes***  All layers | Yes*  All layers |
| VEGFA | No | Yes***  All layers | Yes**  All layers |

Table S1: HC: Healthy controls, AD: atopic dermatitis, NLS: non-lesional skin, LS: lesional skin, T6: Tape 6, T11: Tape 11, T16: Tape 16, T21: Tape 21.

For each column (HC vs AD NLS, HV vs AD LS, AD NLS vs AD LS) a comparison has been made for each depth (T4, T6, T11, T16, T21). Significant findings after adjusting for multiple testing, are marked with “Yes”. * p<0.05, ** p< 0.01, *** p< 0.001. T4: Tape 4,

Test for multiple comparison, non-parametric analysis has been performed, with correction for multiple testing using Dunn’s test. For HC vs AD NLS and AD LS, un-paired analyses has been performed (Kruskal-Wallis), for AD NLS vs AD LS, paired analyses has been performed (Friedman).

Figure S3: Soluble protein content in atopic dermatitis and healthy control skin

Mean protein content in healthy controls (HC) and atopic dermatitis (AD) patients, in lesional skin (LS) and non-lesional skin (NLS). A significant difference was found between AD LS and AD NLS (corrected p-value = 0.02) and between AD Ls and HC (corrected p-value = 0.03). Protein is determined using Pierce BCA protein kit.
